# A one-commodity pickup-and-delivery traveling salesman problem solved by a two-stage method: A sensor relocation application

**DOI:** 10.1371/journal.pone.0215107

**Published:** 2019-04-17

**Authors:** Kun Miao, Hailan Duan, Feng Qian, Ye Dong

**Affiliations:** School of Civil Engineering, Central South University, Changsha, Hunan, China; RMIT University, AUSTRALIA

## Abstract

In the carrier-based coverage repair problem, a single mobile robot replaces damaged sensors by picking up spare ones in the region of interest or carrying them from a base station in wireless sensor and robot networks. The objective is to find the shortest path of the robot. The problem is an extension of the traveling salesman problem (TSP). Thus, it is also called the one-commodity traveling salesman problem with selective pickup and delivery (1-TSP-SELPD). In order to solve this problem in a larger sensor distribution scenario more efficiently, we propose a two-stage approach in this paper. In the first stage, the mature and effective Lin–Kernighan–Helsgaun (LKH) algorithm is used to form a Hamiltonian cycle for all delivery nodes, which is regarded as a heuristic for the second stage. In the second stage, elliptical regions are set for selecting pickup nodes‚ and an edge-ordered list (candidate edge list, *CEL*) is constructed to provide major axes for the ellipses. The process of selecting pickup nodes and constructing the *CEL* is repeated until all the delivery nodes are visited. The final *CEL* stores a feasible solution. To update it, three operations—expansion, extension, and constriction—are applied to the *CEL*. The experimental results show that the proposed method reduces the computing time and achieves better results in higher-dimensional problems, which may facilitate the provision of solutions for more complicated sensor networks and can contribute to the development of effective and efficient algorithms for the one-commodity pickup-and-delivery traveling salesman problem (1-PDTSP).

## 1. Introduction

Wireless sensor networks (WSNs) consist of small nodes with sensing, computation, and wireless communications capabilities that are randomly assigned in monitoring areas. One of the fundamental services provided by WSNs is the monitoring of a specified region of interest. Currently, WSNs are widely used in traffic monitoring, sea resource reconnaissance, military applications, etc. [1]. However, sensors often fail randomly at run time for various reasons such as power depletion or hardware defects, creating unmonitored locations in the given environment. Such locations are often referred to as sensing holes. In order to fill these sensing holes, redundant sensors or previously deployed sensors [2] need to be moved to a particular location, a process that is called sensor relocation [3].

WSNs have attracted considerable research attention for a long period [4–6]. However, the deployment of a large number of mobile sensors is not cost-effective [3]. Moreover, mobile sensor self-relocation [7][8] is not suitable for all situations—for example, a hostile environment, where sensors cannot be manually deployed or air-dropped [4]. However, for these stationary sensors on the WSN, the replacement of damaged sensors with the help of a robot or a group of robots is cost-effective. The number of robots is generally much smaller than the number of sensors. Thus‚ the cost is expected to be lower compared to the use of many mobile sensors.

Furthermore, the robots make full use of redundancy and increase the timeliness of coverage repair with unconstrained movement. For robot-assisted stationary sensors, WSNs can be functionally expanded with the addition of a mobile robot. A WSN with robots performing some sensing task is referred to as a Wireless Sensor and Robot Network (WSRN) [9,10].

The robot-assisted sensor repairing (RASR) [3] problem is presented as a specific WSRN-related problem. The following scenario is considered in this paper. A mobile robot with limited capacity starts from the depot and returns after carrying passive nodes to fill up all the delivery nodes. To save energy, redundant or spare sensors stay in “sleeping” mode, and are thus called passive sensors [11], while nonredundant sensors are referred to as active sensors [12]. When sensors fail or are detected by a Risk Management Framework (RMF) [12] as being vulnerable to a high risk of failure, a single mobile robot with a limited number of sensors is dispatched periodically from a depot. It collects passive sensors over the region of interest as soon as possible and drops the sensors carried from the depot or the passive sensors into the sensing holes where active nodes have failed. As the robot move throughout the environment, they communicate with nearby sensors to determine the location of passive sensors or sensing holes. The mobile robot can choose many trajectories, but it is necessary to find an “optimal” trajectory so that network coverage can be restored by replacing all the damaged sensors. A mobile robot with limited capacity starts from the depot and returns after carrying passive nodes to fill up all the delivery nodes in WSRNs. The problem to find the optimal trajectory of the robot is defined as “the one-commodity traveling salesman problem with selective pickup and delivery” (1-TSP-SELPD) and was presented by Falcon et al. [13]. The original work called it the carrier-based coverage repair problem. Passive sensors are regarded as pickup customers‚ while sensing holes are regarded as delivery customers in 1-TSP-SELPD. This problem relaxes the requirement of visiting all nodes by allowing selectivity of pickup nodes; hence‚ the number of feasible solutions to this problem exceeds that of the TSP problem under the same scale, because this selectivity greatly increases the complexity. For example, the optimal trajectory is produced by ant colony optimization (ACO) and six ACO heuristic functions in [13]. However, this approach is not easy to implement for a larger number of sensors. Large-scale sensor distribution requires more computing power. Thus, the goal of this paper is to develop more effective and efficient algorithms to find the optimal trajectory. Besides, pickup and delivery problems and their variants are a large class of practical real-life problems, which the study of 1-TSP-SELPD will further enrich.

The optimal cycle of the robot is close to the shortest cycle of connecting all the sensing holes to be filled (the sensors to be replaced, i.e., the delivery nodes in the 1-TSP-SELPD) as all the holes must be visited. Thus, we considered solving the problem in two stages: (1) forming a delivery nodes path and (2) constructing a path including pickup nodes and delivery nodes. The goal of the first stage is to obtain a baseline path for the second stage, and the goal of the second stage is to search pickup nodes to construct a sequential edges list with the baseline path. The final list is the solution, i.e., the optimal robot trajectory. To achieve the first goal, all the delivery nodes are linked to a cycle with the Lin–Kernighan–Helsgaun algorithm [14], which exhibits high performance for the TSP [15] with one of the most successful heuristics [14,16]. It is expected to provide good guidance and reduce the difficulty of searching in the second stage. To achieve the second goal, selected pickup nodes are inserted to gradually construct an edges list until a feasible tour is attained. The construction is implemented by expanding, extending‚ or constricting the edges list in a random way, which is quite different from the conventional insertion by adding one node to the tour at a time. The pickup nodes can be selected from pickup nodes sets in a stochastic manner.

The experimental results show that the proposed algorithm can obtain a good robot path for an operation consisting of a single robot repair and replacement of a damaged sensor, and the algorithm is more efficient and effective in solving high-dimensional problems from the standpoint of calculation accuracy and time with low complexity in the second stage with the support of the baseline path.

The main contribution of this paper is to present a new algorithm to solve 1-TSP-SELPD instances. The iterative algorithm works by building an edges list using the baseline path. Three novel operations are proposed to construct the edges list for a solution search. In the process of construction, both the exploration of the solution and the load constraint can be considered. Moreover, the two-stage method is inspired by the heuristic that the optimal cycle is close to the baseline path, which can provide an opportunity to solve larger-scale 1-TSP-SELPD instances.

This paper is organized as follows: Section 2 summarizes the related literature on the robot-assisted sensor relocation problem and the one-commodity traveling salesman problem with pickup and delivery. Section 3 gives a precise definition of the problem. Section 4 gives an overview of the LKH approach. Section 5 describes the algorithm process, including the strategies for selecting pickup nodes and constructing the edges list. Extensive computational results are given in Section 6, and they exhibit good performance on benchmark instances with up to 500 nodes.

## 2. Related work

### 2.1 Robot-assisted sensor relocation problem

Mobile robots were brought into WSNs to provide a wide range of value-added services during the operational lifetime of the networks [17]. In the WSRN, a robot, or a group of robots, is designed to maintain, assist, or optimize the sensor network. The robot adds flexibility not present in a WSN as it does not have too many restrictions. The problem of optimizing the robot trajectory while picking up passive sensors and dropping them at the locations of the damaged sensors in the field has been studied as the RASR problem [9].

In related research, Chang et al. [18] used a robot to achieve distribution, and presented a snake-like deployment algorithm that uses a single mobile robot to deploy static sensors at the vertices of a virtual grid constructed over a bounded region of interest. They require the robot to move to an open area by following a set of predefined rules, dropping a sensor after each step. It is difficult to guarantee full coverage. For full coverage, Belguerche et al. [19] discussed robots that carry redundant sensors to improve the area coverage with a post-deployment after a random deployment.

The carrier-based sensor placement problem [18, 20] first involves the use of robots that carry sensors to replace the damaged sensors. The process requires robots to carry all the sensors at once. However, this is an impractical requirement because each sensor has a limited storage capacity. Falcon et al. [21] proposed a fully distributed scheme and augmentation protocol to allow robots to reuse or repeatedly reload the deployed spare sensors, and they consider the finite robot capacity. However, the sensors’ locations are not random as they are placed on the vertices of an equilateral triangle tessellation. Thus, a novel WSRN scenario in carrier-based coverage repair was identified in the literature [13]. Falcon et al. assumed that one robot is located at a base station and proposed the first centralized solution to the problem by modeling it as a variant of the TSP or vehicle routing problem (VRP). On the basis of the scenario, Falcon and Li et al. [22] further presented a “multiple-carrier coverage repair” problem when a robot team is available. They modeled the WSNs with multi-robot systems as a new generalization of the VRP. A harmony-seeking firefly algorithm was put forward to find the best route plan. Li et al. [11] introduced local communication to discover sensing holes and redundant sensors as the robots moves. They proposed a family of localized robot-assisted sensor relocation algorithms including (1) robots restricted to moving on a virtual grid and (2) those not subject to this restriction. Although in general the grid-base version performs better in shortening the expected traversal time, it imposes grid constraints on robot movement. The second version is more in line with practical needs: the robot moves completely at random. Furthermore, Desjardins et al. [9] regarded the robot trajectory with the shortest length as a multi-objective optimization problem. They took the current energy levels of the passive sensors and their ideal locations into consideration in selecting passive sensors.

### 2.2 The one-commodity traveling salesman problem with pickup and delivery

The one-commodity traveling salesman problem with selective pickup and delivery (1-TSP-SELPD) [13] was proposed and studied by Falcon et al. [13], and there are relatively few other direct studies. However, the similar one-commodity pickup-and-delivery traveling salesman problem (1-PDTSP) has been extensively studied. 1-PDTSP was presented by Hernández-Pérez [23]. In 1-PDTSP, aside from the depot, nodes as customers are partitioned into delivery nodes and pickup nodes. Each delivery node requires a given amount of the product, while each pickup node provides a given amount of the product. One vehicle starts from a depot with a limited capacity, and the products collected by it from the pickup nodes must be supplied to the delivery nodes but not to the depot. By contrast, 1-TSP-SELPD is characterized by the fact that the demand of any delivery customer can be met by a relatively large number of pickup customers [13]. Unlike 1-PDTSP, the passive sensors throughout the field significantly outnumber the sensing holes. Thus, the tour of 1-TSP-SELPD is not a Hamiltonian cycle, in which all delivery nodes (sensing holes) are visited; only some of the pickup nodes (passive sensors) are selected for constructing the optimal tour. Further, the depot provides a certain number of sensors for filling the sensing holes; i.e., the delivery customers’ sensors/commodities can come from the depot and pickup nodes. Moreover, the vehicle returns to the depot with no commodity.

For 1-PDTSP, Hernández-Pérez [23] first presented an exact approach with a branch-and-bound scheme, in which lower bounds are computed by solving a linear program relaxation of the problem. The solving instances can up to 40 customers. After that, they put forward two other heuristic approaches to deal with larger instances in [24]. The first heuristic provides initial upper bounds for their branch-and-cut algorithm with a multi-start greedy search procedure with a k-optimality criterion. The second approach is a branch-and-cut procedure for finding an optimal local solution. Fan Wang et al. [25] studied 1-PDTSP on a path and on a tree topology, respectively. An optimization algorithm in polynomial time was proposed for the path case for various cases of the vehicle capacity k. For the tree case, two optimization algorithms were proposed only for two special cases, when the vehicle capacity is ∞ and 1, but the problem with an arbitrary capacity k was not discussed. Hernández-Pérez et al. further presented a hybrid heuristic method that combines a greedy randomized adaptive search procedure with variable neighborhood descent in [26]. The new heuristic yields better results for larger instances. Zhao et al. [27] presented a genetic algorithm (GA) to solve the problem. In it, 2-opt is used to accelerate the convergence of the GA after a pheromone-based crossover operator constructs offspring by utilizing pheromone trails and some local information including edge lengths and demands of customers to construct offspring. Hosny et al. [28] introduced a new adaptive hybrid variable neighborhood search with a simulated annealing approach. The neighborhood size is adaptive with the current stage of the search in each run. Despite good performance in the large-size test cases, the search requires more processing time. Nenad Mladenović et al. [29] presented a variable neighborhood search approach by k-opt, double-bridge‚ and insertion operators with a binary indexed tree data structure, which improves the best-known solutions of the problem with 200 to 500 customers.

There are also some other similar research problems. Han et al. [30] introduced 1-PDTSP with restricted depot, in which the vehicle departs from and returns to the depot with no goods loaded. They designed an approximation algorithm and a heuristic algorithm to solve large-scale problems. In it, the LKH algorithm proposed by Keld Helsgaun [14][31] is modified to find two Hamiltonian cycles: one traverses all pickup stations‚ and the other traverses all delivery stations. After merging the two TSP tours with a selected merging strategy, the tour is improved by combining the adapting 2-opt and 3-opt heuristics proposed in Lin and Kernighan [32] with the modifications suggested in Savelsbergh [33]. The scatter search algorithm [34] is an evolutionary heuristic for integer programming. Euchi et al. [35] presented a GA hybrid with the scatter search algorithm to solve the 1-commodity pickup-and-delivery VRP with soft time windows by combining a set of diverse and high-quality candidate solutions.

Besides, there are many variants of pickup-and-delivery routing problems described in the literature involving logistics. For example, Mosheiov [36] introduced a TSP with pickup and delivery (TSPPD). In the TSPPD, there are pickup and delivery customers and a vehicle with a given capacity. The objective is to find the minimum length tour for the vehicle when each customer is visited exactly once. The difference between TSPPD and 1-PDTSP is that the former demands that the product collected from pickup customers be delivered only to the depot and the product delivered to the delivery customers always be from the depot, whereas the latter allows the product collected from pickup customers to be delivered to a delivery customer. Another variant of 1-PDTSP is the bike rebalancing problem (SBRP) [37], which involves repositioning bikes among stations in self-service bike-sharing systems. The main difference from 1-PDTSP is that SBRP allows a customer to be visited several times at each station. Moreover, SBRP allows a customer to be used as a temporary depot to temporarily collect and deliver bikes. An increasing number of studies have been performed on the bike rebalancing problem, whether it is an exact method or a heuristic one [37, 38].

1-TSP-SELPD is a typical problem of combinatorial optimization; this means exponential explosion can easily arise when the dimension of the problem becomes high. The algorithm that Falcon et al. designed for 1-TSP-SELPD might face some difficulty in larger-sized problems. Unlike this method, we do not construct a tour node by node, and we think our approach may be more effective in larger-size problems.

## 3. Problem description and mathematical formulation

### 3.1 Problem description

1-TSP-SELPD is a combinational optimization problem, and its mathematical model can be represented by a complete graph $G\;=\;\left(V,E\right),$ where $V\;=\;\left\{V_{0}\;,\;V_{1}\;,\;\ldots ,\;V_{N}\;\right\}\;$ is the vertex set, and $E\;=\;\left\{e_{ij}\;\right\}$ is the edge set. Let N be the total number of customers (nodes excluding the depot).

Consider graph G formed by three kinds of nodes: delivery nodes, pickup nodes, and the depot. Each delivery node represents a sensing hole, which needs to be filled by a sensor. Each pickup node is responsible for providing only one sensor, and the depot can also supply $Q_{0\;}\;$ sensors; i.e., sensors to fill the sensing holes come from both the pickup nodes and the depot. The mobile robot starts from the depot and returns to the depot to form a cycle after filling every sensing hole. The tour is not a Hamiltonian cycle as in the traditional TSP; i.e., all the delivery nodes have to be visited, but not every pickup node need be visited.

Elements in V are either pickup nodes or delivery nodes with demands $q_{i}$ (1 for pick-up nodes and −1 for delivery nodes). Moreover, $V_{0}$ is the depot with $q_{i}\;=\;0.\;e_{ij}$ is the edge between node i and node j, where ∀i,j∈V, and the cost of $e_{ij}$ is equal to its Euclidean distance $d(e_{ij})$. The mobile robot can carry at most $Q_{max\;}$ sensors, and therefore the load l must satisfy $0\;\leq \;l_{ij}\;\leq \;Q_{max}$ when it passes through $e_{ij}$. The objective is to find the shortest tour φ, i.e., $\min\sum_{e_{ij\in \varphi }}d(e_{ij})$ that satisfies the following requirements:

1. The mobile robot starts from and ends at the depot. The load $l_{ij}$ carried from the depot is greater than or equal to 0.
2. The in-degree of the delivery node is equal to the out-degree, which is equal to 1; the in-degree of the pickup node is equal to the out-degree, which is less than or equal to 1.
3. All the delivery nodes have to be filled up.
4. A delivery node can accept only one load, and a pickup node can provide only one load.
5. The mobile robot’s load must be sufficient to satisfy the demand of the delivery node before any visit to a delivery node, and the mobile robot’s load must not exceed the robot’s capacity after any visit to a pickup node.

### 3.2 Problem formulation

The notions of variables are provided below:

$V_{P}$ Set of pickup nodes.
$V_{D}$ Set of delivery nodes.
$V_{0}$ The depot.
V Set of nodes, indexed by i=0,1,…,n with 0 representing the depot.$V=V_{P}\cup V_{D}\cup V_{0}$.
$c_{ij}$ The cost of edge $\left(i,j\right)\in E,\;c_{ij}>0$; a symmetric distance matrix ($c_{ij}$) satisfies the triangle inequality.
$l_{ij}$ The load on the robot when it travels directly from node i to node j; $l_{ij}$ takes an integer value.
$Q_{max}$ The capacity of the mobile robot.
$q_{i}$ The demand at node i. $q_{i}>0$ if $i\in V_{P}\;;\;q_{i}<0$ if $i\in V_{D}$.

Decision variables:

$$x_{ij}=\;\{\begin{array}{c} 1,\;\;\;\;\;\;\;\;\;\;\;\;\;\;\;\;\;\;\;\;\;\;\;if\;tour\;contains\;edge\left(\text{i},\text{j}\right).\\ 0,\;\;\;\;\;\;\;\;\;\;\;\;\;\;\;\;\;\;\;\;\;\;\;\;\;\;\;\;\;\;\;\;\;\;\;\;\;\;\;\;\;\;\;\;\;\;\;\;\;otherwise. \end{array}$$

On the basis of the above notations, 1-TSP-SELPD can be formulated mathematically as follows:

$$\min\underset{i\in V}{\sum }\underset{j\in V}{\sum }c_{ij}x_{ij}\;$$ (1)

$$\text{s}.\text{t}.\;\underset{i\in V}{\sum }x_{ij}=\underset{i\in V}{\sum }x_{ji}\leq 1,\;\forall j\in V_{P}$$ (2)

$$\underset{i\in V}{\sum }x_{ij}=\underset{i\in V}{\sum }x_{ji}=1,\;\forall j\in V_{D}\cup \left\{v_{0}\right\}$$ (3)

$$\;\;\underset{i\in V,i\neq j}{\sum }l_{ji}-\underset{i\in V,i\neq j}{\sum }l_{ij}=q_{j},\;\forall j\in V\backslash \left\{v_{0}\right\}$$ (4)

$$0\leq l_{ij}\leq Q_{max}\;\cdot x_{ij},\;\forall i,j\in V_{}$$ (5)

$$\underset{i,j\in S}{\sum }x_{ij}\leq \left|S\right|-1,\;\forall S\subseteq V\backslash \left\{v_{0}\right\}$$ (6)

$$\;x_{ij}\in \left\{0,1\right\}\;,\forall i,j\in \text{V}$$ (7)

Eq (Eq 1)states the objective of 1-TSP-SELPD, which is to minimize the total travel cost of the mobile robot. Constraints $\left(2\right)$ ensure that each pickup node is visited at most once. Constraints $\left(3\right)$ require that each delivery node and the depot must be visited exactly once. Constraints (4) state that the load change of the robot on the adjacent edge before and after the node is 1 or −1. Constraints $\left(5\right)$ allow the load of each edge to be sufficient to satisfy the demand and not exceed the capacity of the robot. Constraints $\left(6\right)$ eliminate the sub-tours among nodes. Constraints (7) define the decision variables to be binary or non-negative.

## 4. LKH algorithm

In the process of solving for the shortest trajectory, we first look for a Hamiltonian cycle connecting all the sensor holes. This process is called the first stage. Only by finding a correct Hamiltonian cycle can we lay the foundation for the second stage and find the optimal path of the robot.

Lin and Kernighan [32] introduced a heuristic based on the exchange of k tour edges, called k-swap or k-opt. By converting one tour into another in terms of exchange, the intersection of the edge sets of the tours is used to guide further runs. Given a feasible tour, the algorithm repeatedly performs the exchanges that reduce the length of the current tour, until a tour is reached for which no exchange yields an improvement. This process may be repeated many times from initial tours generated in some randomized way.

The Lin–Kernighan heuristic (LK) [32] remains at the heart of the most successful approaches [39], and it is generally considered to be one of the most effective methods for generating optimal or near-optimal solutions for the symmetric TSP. Later on, Helsgaun [14] introduced and implemented lower tolerances (α-values) for an approximation of Held-Karp’s 1-tree with the objective of improving the LK heuristic. Moreover, he presented the Lin–Kernighan–Helsgaun (LKH) algorithm, which is an effective modification of the LK algorithm. The LKH algorithm begins a trial by randomly generating an initial solution, which is iteratively improved using the variable λ-Opt neighborhood function [40]. The modified algorithm uses larger (and more complex) search steps than the LK heuristic. The effectiveness is achieved through an efficient search strategy and sensitivity analysis to direct and restrict the search [14]. The LKH makes it possible to find optimal solutions to large-scale problems.

The LKH algorithm differs in many details from the LK algorithm. For candidate sets, the original algorithm uses the five nearest neighbors of a given city as candidate sets, but Helsgaun introduces α-nearness based on 1-trees as candidate sets. The α-measure can identify those edges that can be included in an optimal tour. Thus, the candidate set may be smaller, but the solution quality is not degraded. For edges movements, the LKH algorithm revises this basic search structure as a sequential 5-opt move, and the moves are sequences of one or more 5-opt moves.

Computational experiments have shown that LKH is highly effective. LKH has produced optimal solutions for all solved problems including a 109399-city instance. Furthermore, the algorithm has improved the best-known solutions for a series of large-scale instances with unknown optima, among these a 1,904,711-city instance [16].

Finding the Hamiltonian cycle of the sensor holes is an independent process and is one stage of a two-stage algorithm in this paper. Therefore, we directly use the computer program [16]. In our work, all the parameters have been set as the default values that Helsgaun had provided. With the program, we need to focus on only the implementation of the second stage.

## 5. Proposed algorithm

In the carrier-based coverage repair problem in wireless sensor and robot networks, passive sensors are pickup customers, and sensing holes are delivery customers. In the following, a “passive sensor” is represented by “p-node,” and a “sensing hole” is represented by “d-node.”

Each damaged sensor needs to be replaced; i.e., the robot should certainly traverse these nodes. Therefore, all d-nodes are the components of a feasible solution path, which the robot must walk through. On the other hand, in these passive sensors (p-nodes), those p-nodes that are closer to the shortest cycle connecting all the d-nodes (called the LKH cycle) have greater chances of being the nodes of the shortest 1-TSPSELPD path. Thus, the LKH cycle should be the cycle that the robot expects to approach. The LKH cycle may be regarded as heuristic knowledge of the optimal solution (i.e., the shortest cycle that the robot traverse, called the R-cycle). With the heuristic, we developed a two-stage method to find the R-cycle. The first of the two stages is building the shortest Hamiltonian cycle (LKH cycle) of all the d-nodes, which is a TSP and is constructed by the LKH algorithm in Section 4.

**Fig 1:**
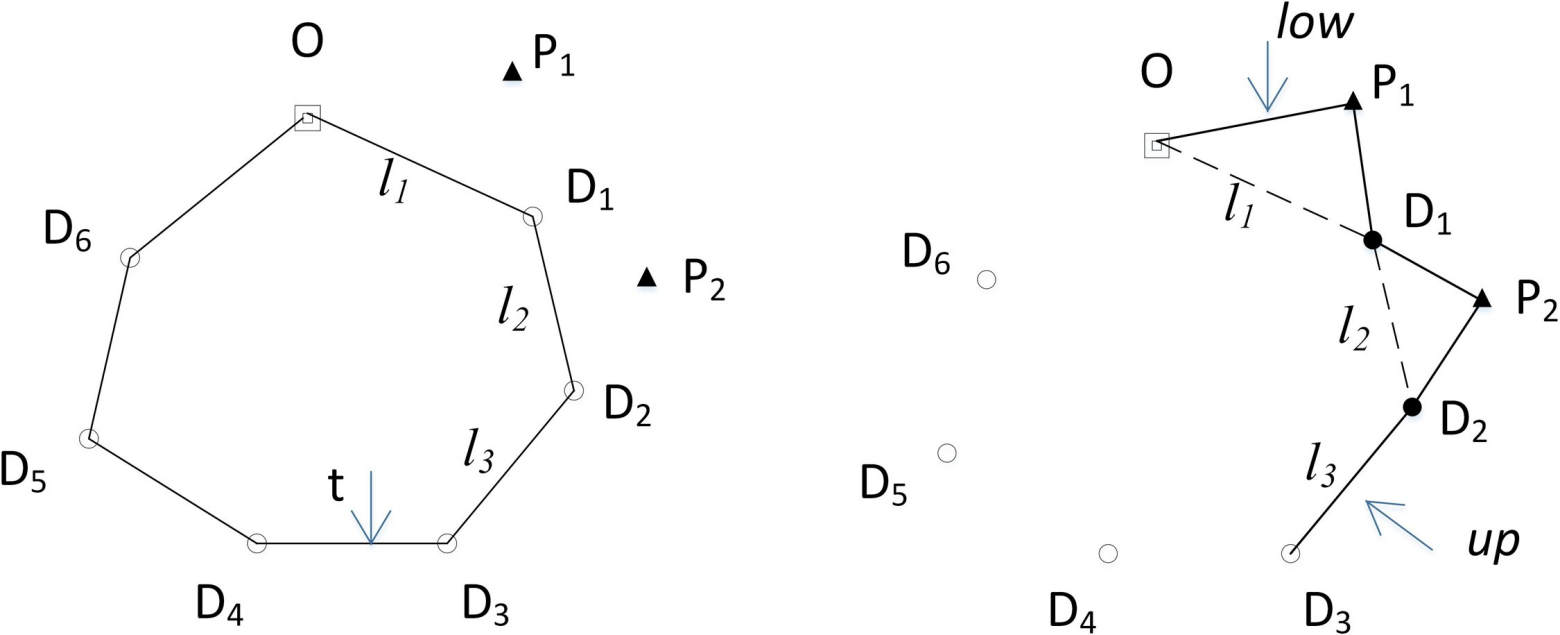
(a) LKH cycle ($L_{LKH}$). (b) Edges 1 to t-1 in $L_{LKH}$ in Fig 1A are moved into the *CEL*, and the *CEL* is expanded after p-nodes ${\text{P}}_{1}$,${\text{P}}_{2}$ are selected and inserted into the R-path (squares represent the base station, triangles represent p-nodes, hollow circles represent d-nodes to be filled, and solid circles represent filled d-nodes).

The LKH edge is defined as the edge connecting any adjacent two d-nodes in the LKH cycle. The edge is denoted by *l*_*i*_(*i* = 1,2,…,*N*_*d*_+1), where *N*_*d*_+1 is the number of edges of the cycle (i.e., the number of all the d-nodes is *N*_*d*_, not including the base station); and *L*_*LKH*_ = {*l*_1_,*l*_2_,…,*l*_*Nd*+1_}, where *L*_*LKH*_ denotes the ordered list of all the LKH edges (e.g., the edges in Fig 1A).

However, the LKH cycle is an incomplete tour for the robot. This tour will be further expanded by adding p-nodes using a random search method proposed in the second stage. In other words, the LKH edges will be supplied to construct an R-cycle as auxiliary edges in the second stage.

**Fig 2:**
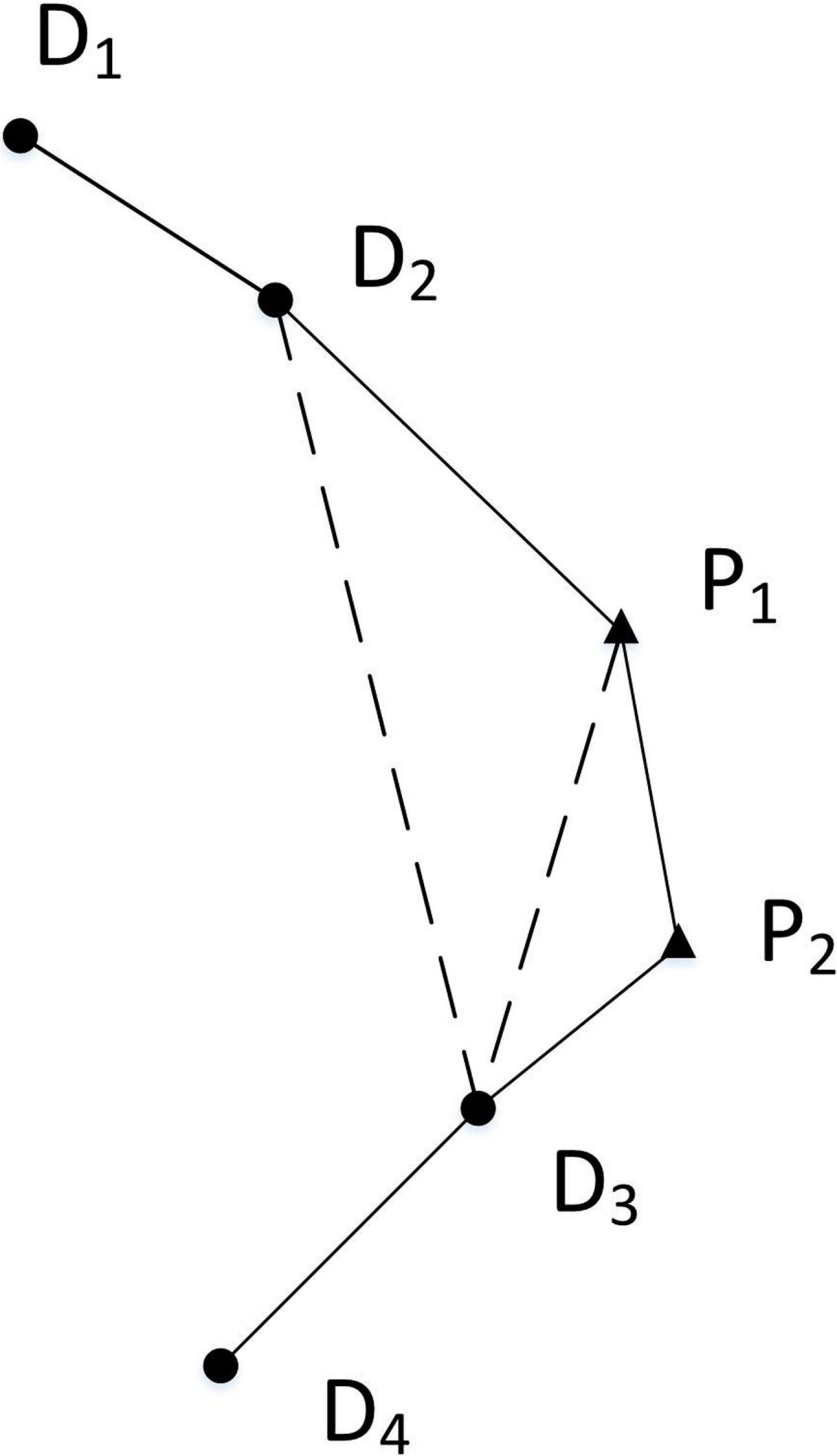
A p-node is inserted near the edge in *CEL* to form a new path (triangles represent p-nodes, and circles represent d-nodes).

In the second stage, the construction of the R-cycle includes two processes: (1) selecting edges: build a CEL (a path is represented as a list of edges in this paper), then select one edge in this list and (2) selecting nodes: select a p-node near the selected edge. For example, in Fig 2,${\text{D}}_{\text{1}}$,${\text{D}}_{2}$,${\text{D}}_{3}$, and ${\text{D}}_{4}$ are the d-nodes to be filled, and the edges formed by connecting these d-nodes have been incorporated into the *CEL*. In the *CEL*, p-node ${\text{P}}_{\text{1}}$ is selected in the vicinity of the edge ${\text{D}}_{\text{2}}{\text{D}}_{\text{3}}$ (within a certain range) after this edge is selected, and then this edge is deleted. Thus‚ a new local path D1DP21D3D4 is formed. That is, the CEL is updated to D1DP21D3D4. Besides, continuous selection of edges and nodes is allowed. For example, in Fig 2, edge ${\text{P}}_{\text{1}}{\text{D}}_{\text{3}}$ is selected in the updated CEL, and then node ${\text{P}}_{\text{2}}$ in the vicinity of the edge ${\text{P}}_{\text{1}}{\text{D}}_{\text{3}}$ is selected, ${\text{P}}_{\text{1}}{\text{D}}_{\text{3}}$ is deleted, and an updated path ${\text{D}}_{\text{1}}{\text{D}}_{\text{2}}{\text{P}}_{\text{1}}{\text{P}}_{\text{2}}{\text{D}}_{\text{3}}{\text{D}}_{\text{4}}$ is formed.

The LKH cycle formed by the $N_{d}$ d-nodes and the base station contains $N_{d}+1$ edges. Every time a p-node is inserted into the cycle, an edge is added in the path. The total number of edges of the final R-cycle is $2N_{d}-q_{0}+1$, where $q_{0}$ is the number of loads carried by the robot from the base station; i.e., the total number of elements of the final *CEL* is $2N_{d}-q_{0}+1$. The reasons are as follows:

1. When the robot does not carry loads from the base station (i.e., $q_{0}\text{=}0$), $N_{d}$ loads at the p-nodes are selected to be delivered to equal numbers of d-nodes; i.e., $N_{d}$ edges should be added to the *CEL*. Therefore, the total number of elements of the *CEL* is $2N_{d}+1$.
2. When the robot carries $q_{0}$ loads from the base station, the $q_{0}$ loads are first delivered to the $q_{0}$ d-nodes. Thus, only $N_{d}-q_{0}$ loads from the p-nodes are required to be picked up. Accordingly, $N_{d}-q_{0}$ edges will be inserted into the R-cycle. Therefore, the number of elements of the *CEL* is $\left(N_{d}+1)+(N_{d}-q_{0}\right)$, or $2N_{d}-q_{0}+1$.

### 5.1 Construction of ellipse sets—selection of p-nodes

Which p-nodes should be selected for building the *CEL*? The method here is to randomly select an edge e in the *CEL*, and then randomly select a p-node in the region determined by the edge. This process is called selecting a p-node in an ellipse set. The basic principle of selecting a p-node is to minimize the length of the path formed by the insertion of the node. The simplest selection method is to choose the node that produces the minimum sum of the distances from it to the two endpoints of the edge e. However, the unique p-node that satisfies the request is selected around edge i. Hence‚ other p-nodes have no chance of being chosen, and no feasible solution is explored. However, a combination of different p-nodes may produce a more feasible solution to make the path shorter.

**Fig 3:**
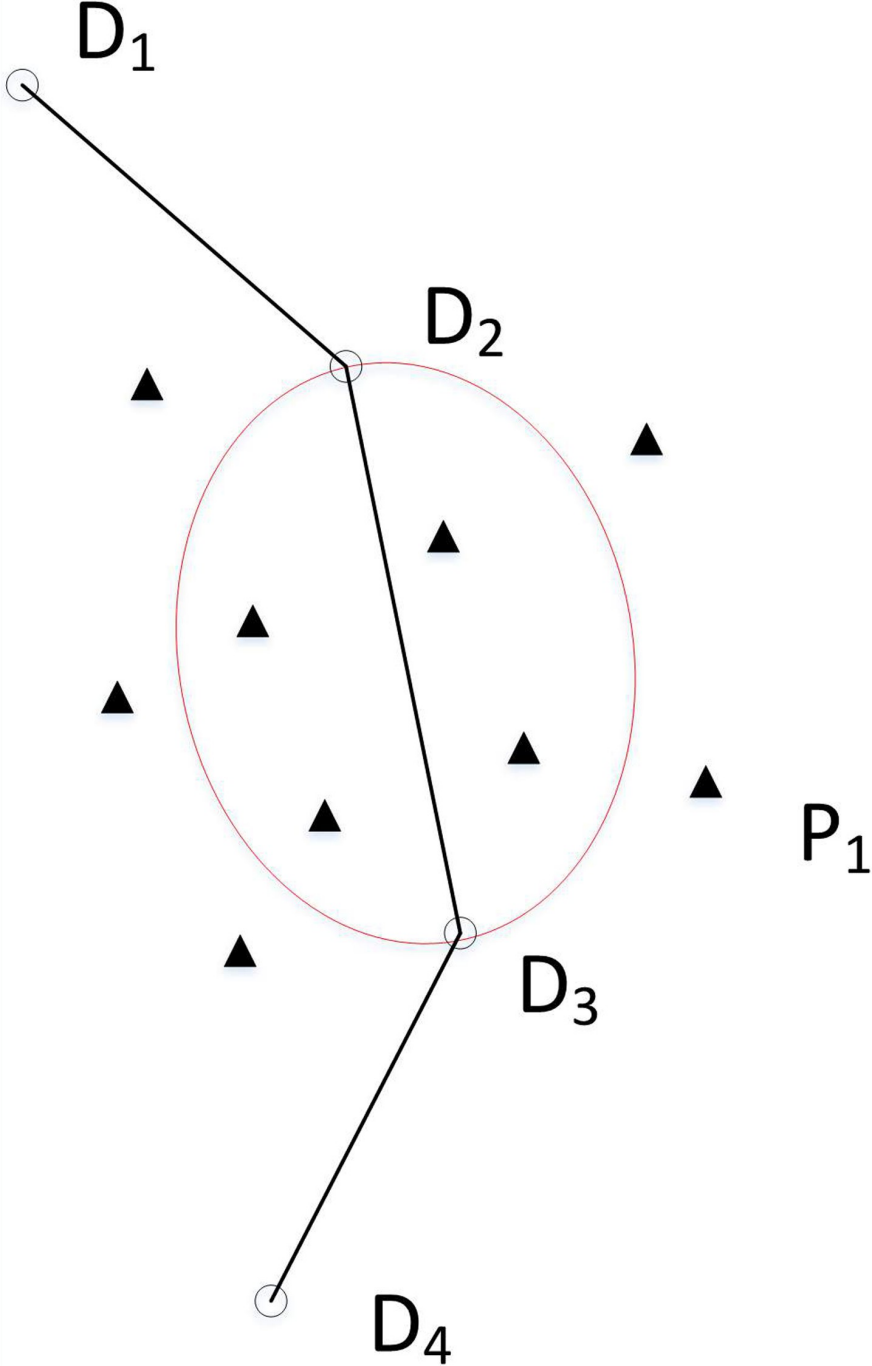
Elliptical region (hollow circles represent d-nodes to be filled, and triangles represent p-nodes).

An elliptical region based on the selected edge e (Fig 3) is presented to select a favorable p-node to explore the combination of p-nodes that will enable a feasible solution/path to be constructed. The p-nodes in the region are all near the selected edge e, and therefore they are candidates to traverse for the robot.

The ellipse set is the set of all the p-nodes in an elliptical region. Any one of the edges on a path is taken as the major axis, and the minor axis is determined by the ratio of the minor axis to the given major axis. The nodes within the scope of the ellipse are the candidate nodes for the optimal path.

The center of the ellipse coincides with the origin of a local coordinate system, UO′V for the standard equation of an ellipse. However, the positions of the d-nodes and the p-nodes are represented in a global coordinate system, XOY. Thus, the ellipse equation is converted from the local coordinate system to the global coordinate system for convenience. If p-node P(x, y) in the coordinate system XOY satisfies inequality (8), it means that the node is in the elliptical region:

$$Loc\left(x,y\right)=\frac{{\left(xcos\alpha +ysin\alpha -S\right)}^{2}}{a^{2}}+\frac{{\left(ycos\alpha +xsin\alpha -T\right)}^{2}}{b^{2}}\leq 1$$ (8)

a=‖e‖/2 (9)

b=a⋅R (10)

where α is the angle at which the global coordinate system is rotated to the local coordinate system; (S,T) is the origin O′ of UO′V in XOY; a is the semi-major axis of the ellipse; b is the semi-minor axis of the ellipse; and R is the ratio of the minor axis to the major axis.

Any one of the nodes in an ellipse has a chance of being selected. Selection acts as a driving force for promising results in the search space. How to select a p-node to obtain an R-cycle easily for sensor replacement is discussed in the following.

Because the LKH has already been operated in the first stage, the complexity of the problem has been reduced, we are not going to adopt a more complicated tool. Roulette wheel selection [41] is most frequently and commonly used in GAs due to its straightforward interpretation and simplicity of implementation [42]. The selection can provide slightly better results despite being somewhat slower than the tournament method [43]. The basic strategy is that the higher the fitness of a solution, the greater the probability of survival, because the probability of selection is proportional to the fitness of an individual in the selection. Thus, roulette wheel selection is here used to grab one p-node for constructing a path. The selection probability for a p-node is kept proportional to its fitness value in the process. Given a p-node ${\text{P}}_{i}$, whose fitness value is given by $f(P_{i})=\frac{1}{\left\|e_{1}{}^{i}\right\|+\left\|e_{2}{}^{i}\right\|-\left\|e^{i}\right\|}$, the probability of selecting ${\text{P}}_{i}$ is

$$p(P_{i})=\frac{f(P_{i})}{\sum_{i=1}^{N_{e}{}^{P}}f(P_{i})},\;(i=1,2,\ldots ,N_{e}{}^{P}).$$ (11)

where $f(P_{i})$ is the fitness of node ${\text{P}}_{i}$,$\left\|e_{1}{}^{i}\right\|,\left\|e_{2}{}^{i}\right\|$ is the distance between the end points of the edge e from $P_{i}$, $\left\|e^{i}\right\|$ is the length of the edge e, $p(P_{i})$ is the selection probability of ${\text{P}}_{i}$, and $N_{e}{}^{P}$ is the number of p-nodes in the ellipse set of the edge e.

If the node with the minimum $\left\|e_{1}{}^{i}\right\|+\left\|e_{2}{}^{i}\right\|-\left\|e^{i}\right\|$ is selected all the time, suboptimal solutions will be produced. Hence, the random method is necessary to mitigate the burden imposed by the fact that selection is controlled by nodes with high fitness. However, the random selection samples only some of the nodes, which may not slow down the search speed.

The above choice is for the case where there is more than one p-node in the ellipse, which is a common occurrence. However, it is possible that no p-node is present in the ellipse. When this occurs, the algorithm selects other edges to construct another ellipse, in which a p-node can usually be obtained with a specified number of iterations; otherwise, it chooses the p-node with the smallest Loc(x,y).

### 5.2 Construction of *CEL*

#### 5.2.1 Basic construction process

The intermediate path for the construction of the R-cycle is called the **R-path**. The R-path is stored in a *CEL*, which is a permutation of the edges. The *CEL* corresponds to the R-path, and is also a dynamic component of a feasible solution. For example, the solid lines in Fig 1B are edge elements in the *CEL*. The R-path finally becomes an R-cycle as the number of edge elements increases.

The edge elements of the *CEL* come in two ways.

1. From $L_{LKH}$. The cycle in Fig 1A is an LKH cycle, and the list corresponding to this cycle is $L_{LKH}$. The edge in the *CEL* coming from $L_{LKH}$ is called an L-edge. In $L_{LKH}$, let t point to the edge that is about to be appended to the *CEL*. Edge t in $L_{LKH}$ is called the **current LKH edge**. In Fig 1A, the pointer t points to the current LKH edge $D_{3}D_{4}$ after $l_{3}$ and its previous edges $l_{1}\sim l_{\text{2}}$ (i.e., $l_{1}\sim l_{t-1}$) have been added to the CEL in Fig 1B.
2. Select a p-node, and then insert it into the R-path to form two adjacent edges at the node. The two edges are added to the with the insertion, which is called an E-edge. In Fig 1B, $OP_{1}$,$P_{1}D_{1}$,$D_{1}P_{2}$, and $P_{2}D_{2}$ are E-edges with ${\text{P}}_{\text{1}}$ and ${\text{P}}_{\text{2}}$ inserted into the R-path.

Any one of *L*-*edge* and *E*-*edge* in the *CEL* is used as the major axis of the ellipse. For example, in Fig 1B, p-node P1 is selected from an ellipse set constructed with the major axis $l_{1}$.P2 is selected like P1 from an ellipse set constructed with the major axis $l_{2}$.

The *CEL* is dynamic, which means that edge elements in it are constantly changing. For example, in Fig 1B, $CEL=\{OD_{1},D_{1}D_{2},D_{2}D_{3}\}$ turns into $CEL=\{OP_{1},P_{1}D_{1},D_{1}D_{2},D_{2}D_{3}\}$ after P1 inserts the *CEL*, then $CEL=\{OP_{1},P_{1}D_{1},D_{1}P_{2}\text{,}P_{2}D_{2},D_{2}D_{3}\}$ after P2 inserts it. low and up, respectively, point to the positions of the start and the end edges of the *CEL* with the edges brought into the *CEL*.

#### 5.2.2 Construction rules of *CEL*

The following rules should be followed for constructing a *CEL*:

1. The tail node of the last edge in *CEL* must be reserved for being filled (a tail node of an edge is a d-node at the far end of an edge from the base station).

One p-node can provide only one load to be picked up. Once one load is picked up at a p-node, a d-node will be filled up. As a result, only one d-node to be filled is reserved in the *CEL* for the load picked up at the p-node. Moreover, this d-node to be filled is specified in the tail node of the last edge in the *CEL* in the algorithm.

1. Edge load constraints.

When one load is picked up at a p-node, the load on the following edge of the p-node is increased by one. On the other hand, the capacity of a robot is the maximum load that it can carry. The edge load is defined as the number of loads carried by the robot as it passes through an edge. Thus, the edge load of an edge in the *CEL* will first reach its capacity with continued picking up of loads on different p-nodes.

The following describes the construction process of the *CEL*.

#### 5.2.3 Initialization of *CEL*

**Fig 4:**
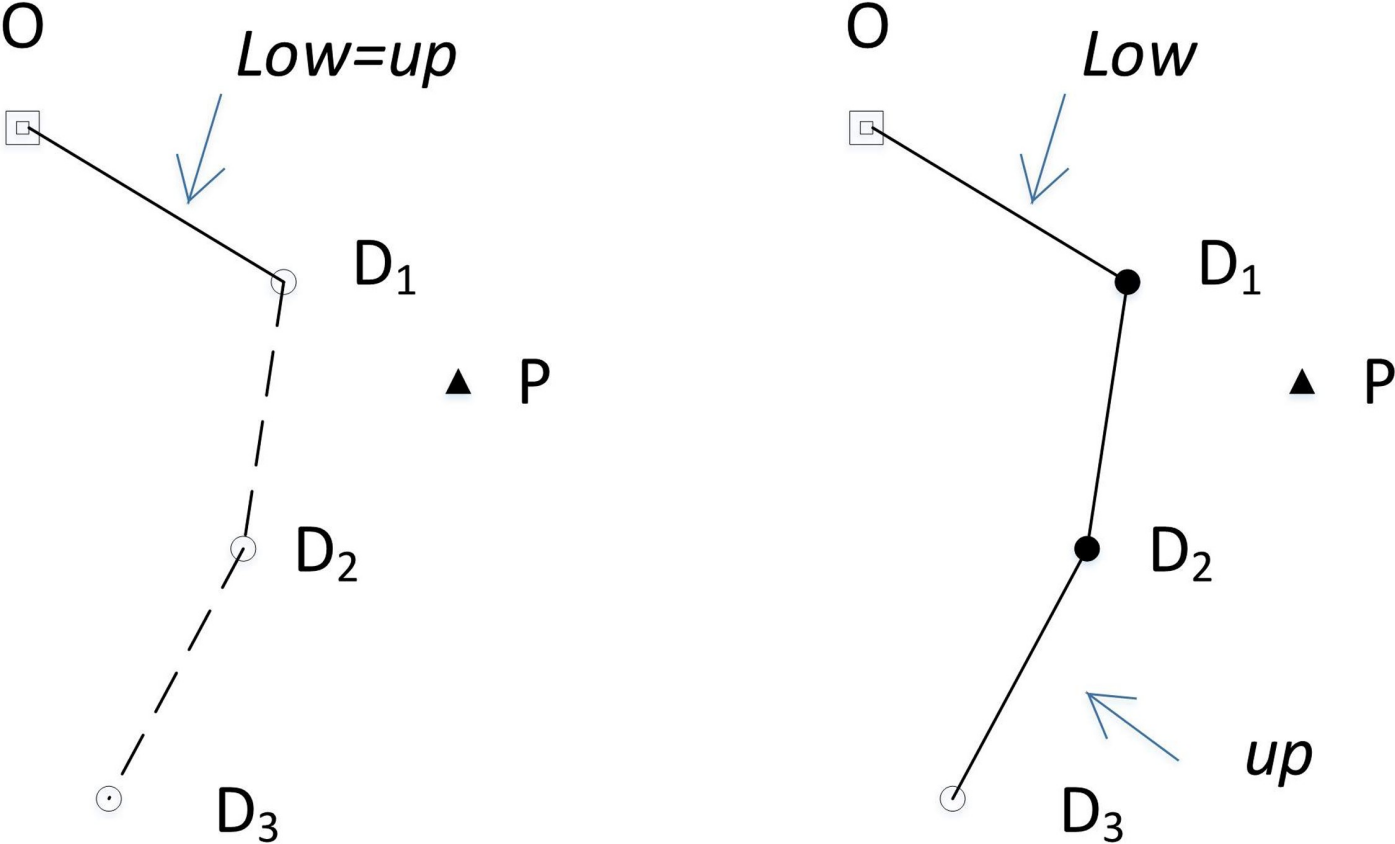
Initialization of *CEL*. (a) $q_{0}=0$. (b) $q_{0}=2$ (squares represent the base station, triangles represent p-nodes, hollow circles represent d-nodes to be filled, and solid circles represent filled d-nodes).

1. $q_{0}=0$; i.e., the base station has no load available. Because one d-node to be filled is always required to be reserved on the path, the first edge ($l_{1}$) of the $L_{LKH}$ is incorporated into the *CEL* for finding a p-node to supply one load, which then can be delivered to the d-node. For example, the first edge of the *CEL* (the solid line in Fig 4A) come from $L_{LKH}$, and t = 2 denotes that the edge about to be incorporated to $L_{LKH}$ is the next edge of ${\text{OD}}_{1}$ in the $L_{LKH}$, i.e.,${\text{D}}_{1}{\text{D}}_{2}$ in the *CEL*.
2. $q_{0}\neq 0$; i.e., the robot carries $q_{0}$($q_{0}\neq 0$) loads from the base station. The first $q_{0}$ edges in $L_{LKH}$ are appended to the *CEL* for providing $q_{0}$ d-nodes. The $q_{0}$ loads are first used to fill the $q_{0}$ tail nodes of the L-edges starting from the base station, which is because the LKH path itself is the shortest path. After the loads fill the $q_{0}$ tail nodes of the first $q_{0}$ L-edges, let $t=2+q_{0}$. Suppose $q_{0}$ = 2 in Fig 4B; ${\text{OD}}_{\text{1}}$,${\text{D}}_{1}{\text{D}}_{2}$ are appended to the *CEL*, and ${\text{D}}_{1}$ and ${\text{D}}_{2}$ are filled with the two carried loads. Another LKH edge is then appended from $L_{LKH}$ to the reserved d-node (${\text{D}}_{\text{3}}$). In Fig 4B, ${\text{D}}_{\text{3}}$ is reserved with the edge ${\text{D}}_{2}{\text{D}}_{3}$ in $L_{LKH}$ being appended to the *CEL* after ${\text{D}}_{1}$ and ${\text{D}}_{2}$ are filled. Let t = 4, which denotes that the edge about to be copied to the CEL is the next edge of ${\text{D}}_{\text{2}}{\text{D}}_{\text{3}}$ in $L_{LKH}$.

The update of the *CEL* can generate more feasible solutions. For this update, three operations—Expansion, Extension, and Constriction—have been proposed to update the *CEL*.

#### 5.2.4 Expansion of *CEL*

There are two sources of loads to fill the d-nodes (i.e., *L-edge*'s tail node): (1) the loads from the base station, which are carried to fill the first $q_{0}$ edges of the *CEL*; (2) the loads obtained by expanding *L-edge* or *E-edge*, which are used to fill the tail node of the last edge of the current *CEL*. Expanding L-edge and E-edge is called expansion, which refers to the process of constructing an ellipse whose major axis is *L-edge* or *E-edge* in the *CEL*, and then selecting one of the p-nodes in the ellipse set and inserting it into the *CEL* to form two new *E-edges*.

**Fig 5:**
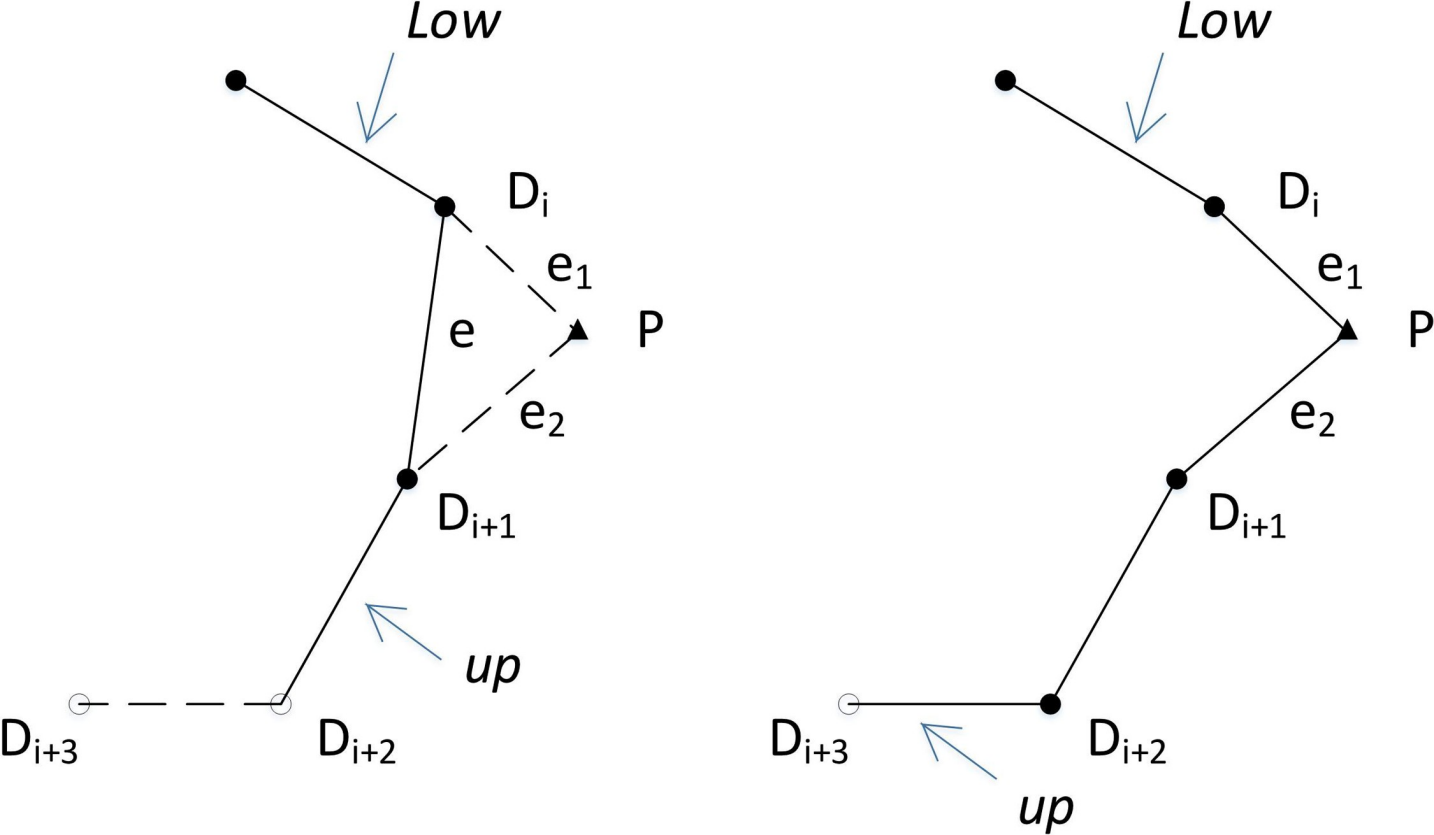
Expansion. (a) Expand $e_{1}$ and $e_{2}$ based on edge e. (b) The path after being expanded (triangles represent p-nodes, hollow circles represent d-nodes to be filled, and solid circles represent filled nodes).

The major axis of the ellipse comes from one edge of the *CEL*, and the edge is selected by the roulette method. An ellipse set is then constructed based on this edge, and a p-node P is obtained in the ellipse set according to the method in Section 5.1. For example, in Fig 5A, edge $e_{1}$,$e_{2}$ (i.e., ${\text{D}}_{\text{i}}\text{P}$,${\text{PD}}_{i+1}$) is added to the *CEL*, and edge e is deleted from the *CEL*, forming the new path shown in Fig 5B.

**Fig 6:**
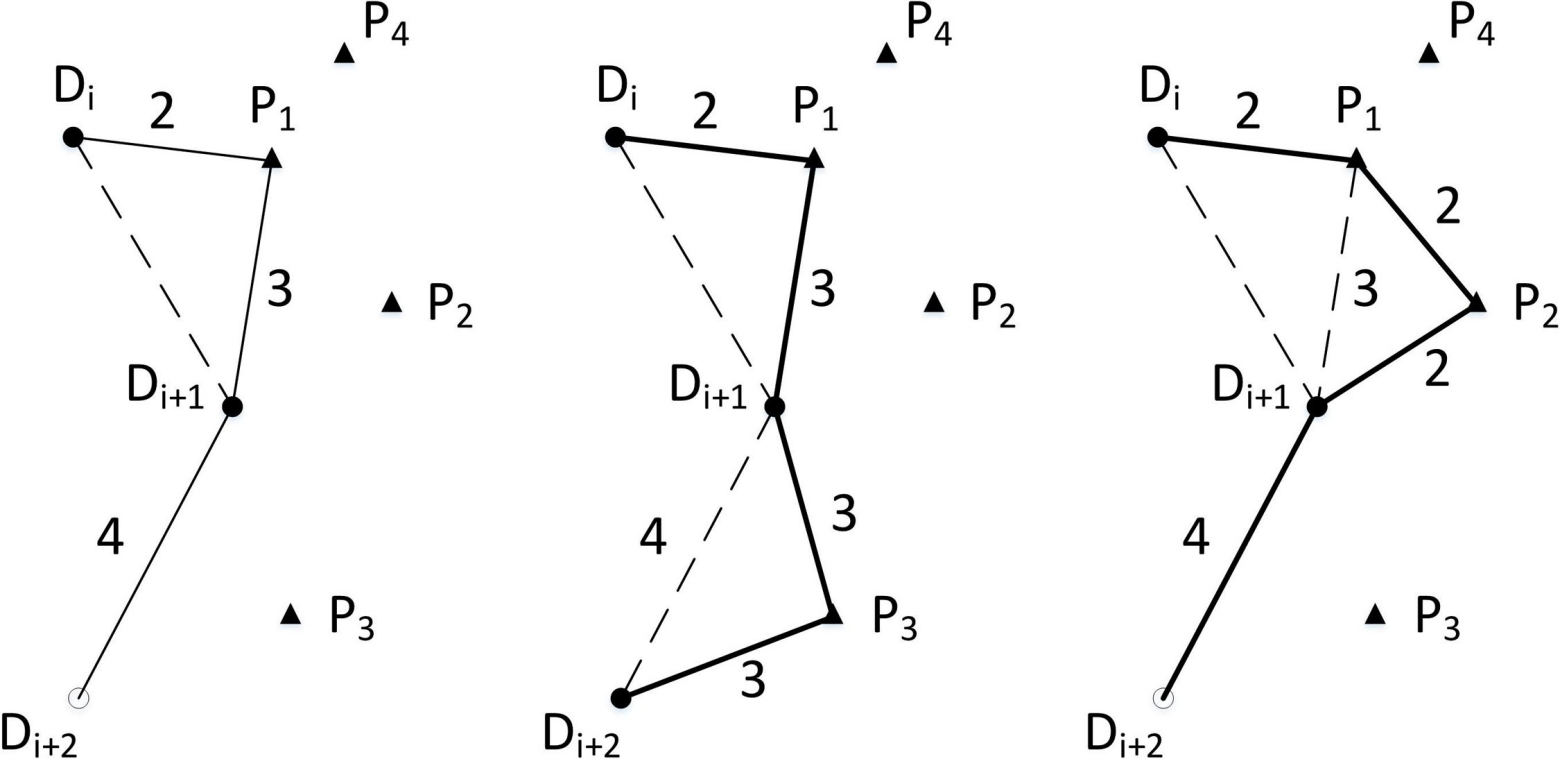
Lengths of the paths are different for different expansions. (a) Expand on *L-edge* ${\text{D}}_{\text{i}}{\text{D}}_{\text{i+1}}$. (b) Expand on *L-edge* ${\text{D}}_{\text{i+1}}{\text{D}}_{\text{i+2}}$. (c) Expand on *E-edge* ${\text{P}}_{\text{1}}{\text{D}}_{\text{i+1}}$ (the number represents the length of the edge, triangles represent p-nodes, hollow circles represent d-nodes to be filled, and solid circles represent filled nodes).

Either *L-edge* or *E-edge* can be expanded. In Fig 6, there are two ways to expand the path ${\text{D}}_{\text{i}}{\text{P}}_{\text{1}}{\text{D}}_{\text{i+1}}{\text{D}}_{\text{i+2}}$(Fig 6A): One method is based on *L-edge ${\text{D}}_{\text{i+1}}{\text{D}}_{\text{i+2}}$*(Fig 6B), in which the edges ${\text{D}}_{\text{i}+1}{\text{P}}_{3}$ and ${\text{P}}_{3}{\text{D}}_{\text{i}+2}$ can be expanded; the other method is based on *E-edge* ${\text{P}}_{\text{1}}{\text{D}}_{\text{i+1}}$ (Fig 6C), in which the edge ${\text{P}}_{\text{1}}D_{i+1}$ can be expanded to ${\text{P}}_{\text{1}}{\text{P}}_{\text{2}}$ and ${\text{P}}_{2}{\text{D}}_{\text{i+1}}$. Furthermore, the latter path length (10 units) is shorter than the former (11 units) in the two cases. Hence, if the latter is selected, it is advantageous for obtaining the optimal solution.

Any edge in the *CEL* can be continuously expanded to obtain more p-nodes and shorten the path. As shown in Fig 6C, ${\text{D}}_{i}{\text{D}}_{i+1}{\text{D}}_{i\text{+2}}$ is the original LKH cycle. After ${\text{P}}_{\text{1}}$ is expanded on ${\text{D}}_{\text{i}}{\text{D}}_{\text{i+1}}$, ${\text{P}}_{\text{2}}$ is further expanded on ${\text{P}}_{\text{1}}{\text{D}}_{\text{i+1}}$. This continuous expansion of *E-edge* is the source of a feasible solution, which widens the scope of the search for p-nodes and increases the diversity of solutions. The termination condition for this continuous expansion occurs when the loads on an edge of the *CEL* reach the capacity of its edge load.

#### 5.2.5 Extension of *CEL*

**Fig 7:**
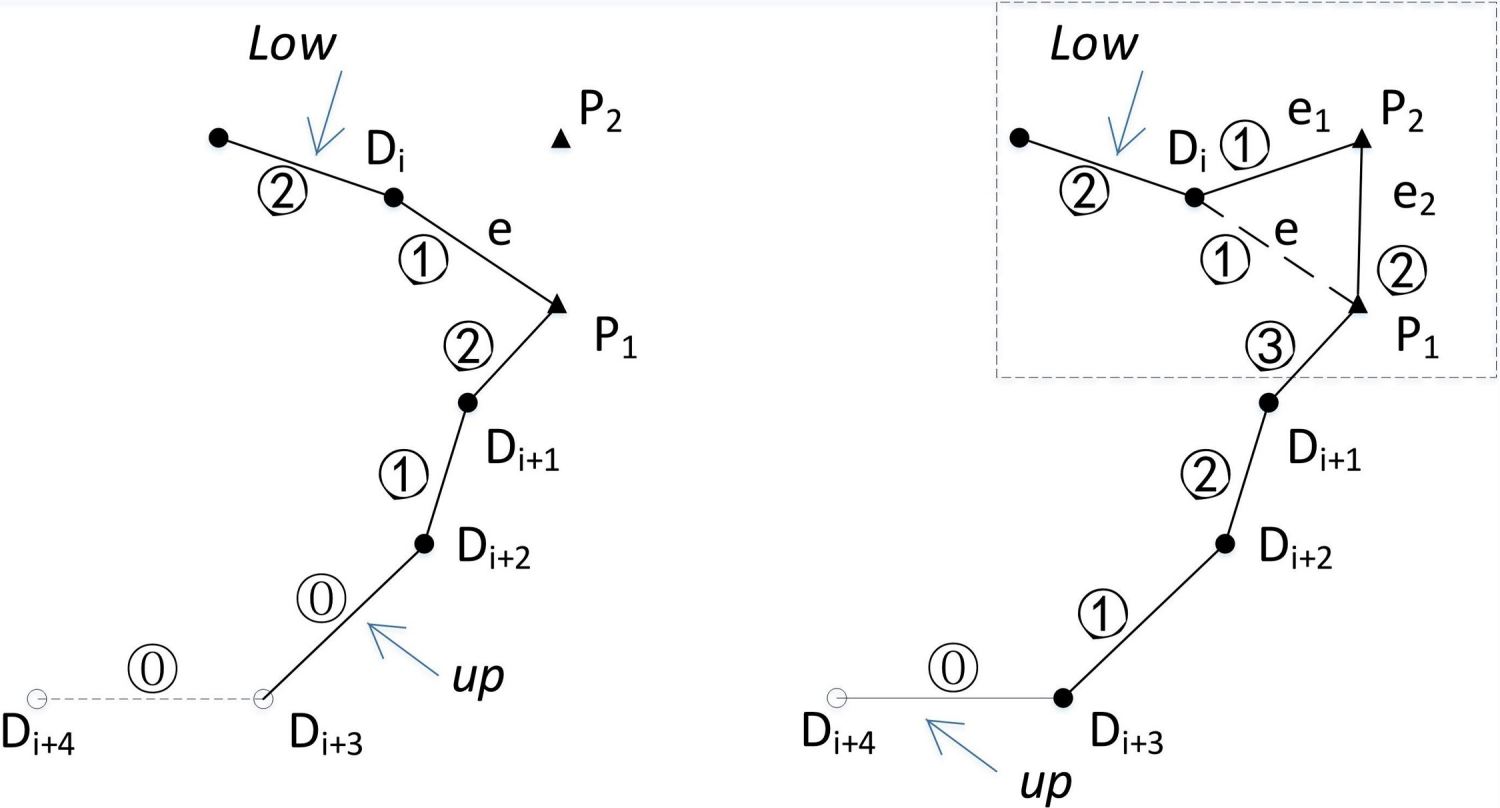
Extension and constriction of the *CEL*, and load changes on edges after picking up one load. (a) Extension, (b) constriction. (The circled number represents the number of loads; i.e., $load_{i}$ represents the load carried by the robot as it passes edge *i*; the dashed line in Fig 7A represents the LKH edges that have not been appended to the *CEL*; low and up, respectively, point to the first and last edges in the *CEL*. Triangles represent p-nodes, hollow circles represent d-nodes to be filled, and solid circles represent filled nodes.).

Every time an expansion is performed on the R-path—i.e., a load is picked up with the expansion—one sensing hole on an edge of the *CEL* must be filled with the load. Thus, the tail node of the last edge of the *CEL* is specified to accommodate the load. The current LKH edge of $L_{LKH}$ (edge t in Fig 1A) is appended to the last edge of the R-path so as to provide a d-node, which extends the R-path and increases the number of edges of the R-path. This process is called extension. In Fig 7A, ${\text{D}}_{i+3}$ will be filled after picking up one load at node ${\text{P}}_{\text{2}}$. The current edge of $L_{LKH}$ (${\text{D}}_{i+3}{\text{D}}_{i+\text{4}}$ in Fig 7A) is then appended to the *CEL* as its last edge; i.e., edge ${\text{D}}_{i+3}{\text{D}}_{i+\text{4}}$ is extended in the *CEL*. Let *up* point to ${\text{D}}_{i+3}{\text{D}}_{i+\text{4}}$, and let its edge load be $load_{up}$; then $load_{up}=\;load_{up-1}-1$, where *low* and *up* represent pointers to the first and last edges of the *CEL*, respectively.

#### 5.2.6 Constriction of *CEL*

Picking up a load will cause edge load changes on the subsequent edges of the pickup node. For example, the edge load on edge $e_{2}$ is one more than that on the previous edge $e_{1}$, i.e., $load_{e_{2}}=load_{e_{1}}+1$, after inserting p-node ${\text{P}}_{\text{2}}$ (Fig 7B). The edge load of each edge following edge $e_{2}$ is increased by one, i.e., $load_{e_{i}}=load_{e_{i}}+1$,$e_{i}\in \{e_{3},\ldots ,e_{up}\}$, where $e_{3}$ represents the edge followed by edge $e_{2}$, and $e_{up}$ represents the last edge of the *CEL*. Fig 7B shows the change in loads on each edge after one load is picked up at node ${\text{P}}_{\text{2}}$.

The expansion will continually increase the loads on each edge in the *CEL*. Thus‚ some edges’ load will reach the capacity. Suppose that the load on one edge k first reaches the capacity; then edge k and its previous edges can no longer be selected to construct ellipse sets. Otherwise, these edges’ loads will violate capacity constraints once a load is picked up. Therefore, edge k and its previous edges have to be cut from the *CEL*, which is called constriction. For example, in Fig 7B, edge ${\text{P}}_{\text{1}}{\text{D}}_{\text{i+1}}$ and its previous edges are removed from the *CEL* when the load of the robot reaches 3 on passing the edge ${\text{P}}_{\text{1}}{\text{D}}_{\text{i+1}}$ with $Q_{\max}=3$. Those being removed edges from the *CEL* are indicated inside the dotted box.

#### 5.2.7 Edge selection in *CEL*

The selection of edges in the *CEL* is also performed by the roulette method. The longer edge in the *CEL* has a larger elliptical area, which may cover more p-nodes. Therefore, the longer edge should be given a greater selection probability when no special sensor distribution rules are given. Let the fitness of edge $e_{i}$ in *CEL* be $g(e_{i})$:

$$g(e_{i})={\left\|e_{i}\right\|}^{M}$$ (12)

The selection probability of $e_{i}$ is

$$p(e_{i})=\frac{g(e_{i})}{\sum_{i=low}^{up}g(e_{i})}$$ (13)

where ${\left\|e_{i}\right\|}^{M}$ represents the length of edge $e_{i}$ raised to the power *M*, and the value of *M* has a certain influence on the result (see Section 6.1).

## 5.3 Algorithmic process

The key to obtaining an optimal path is to build the P-path (*CEL*). A complete *CEL* is a feasible solution. N feasible solutions are obtained with N running times, in which the shortest one is regarded as the best path.

The expansion includes selecting edges and selecting nodes: the edge is selected from the *CEL*, the ellipse set is constructed, a p-node is selected from the ellipse set of the edge, and the new edge $e_{1}$,$e_{2}$ is obtained by inserting the p-node (see Fig 5). Algorithm 1 is to construct the *CEL*, algorithm 2 is to select a p-node in an ellipse set, and Table 1 explains the notations. A run of algorithm 1 can obtain a feasible solution. Algorithms 1 and 2 are described as follows.

**Table 1:** Notations used in algorithms 1 and 2 (also see Fig 7).

*CEL*	A sequence of edges list composed of L-edge and E-edge, where each
	element (edge) may be selected to construct different ellipse sets.
Low,up	Pointers to the first and last edges of *CEL*, respectively.
$q_{0}$	The number of loads carried from the base station.
$L_{LKH}[j]$	Edge j on LKH cycle, $j=1,\ldots \ldots ,N_{d}+1$.
$N_{d}$	The total number of d-nodes, i.e., the total number of LKH edges is $N_{d}+1$.
$N_{d}-q_{0}$	The total number of visited or inserted p-nodes.
$Q_{\max}$	The load capacity of the robot.
$Load_{\left[i\right]}$	The edge load when the robot passing through edge i in *CEL*.

**Algorithm 1.** Constructing *CEL*.

1:    Step 1:    low = 1; up = $q_{0}$+1;$S_{tabu}$ = Null;

2:                    //and up are two pointers to the start edge (low = 1) and the end edge

3:                    (up = $q_{0}$+1) of the *CEL*, respectively; $S_{tabu}$ is a taboo table, i.e., the set of

4:                    p-nodes that have been selected to avoid being selected again.

5:    Step 2:    copy $L_{LKH}[1\sim 1+q_{0}]$ to CEL[low~up];

6:                    // initialization on CEL

7:    Step 3:    $n_{pick}\text{=1}$;

8:                    //$n_{pick}$ is the current number of inserted p-nodes.

9:    Step 4:    $t=2+q_{0}$;

10:                    //t points to the current edge of $L_{LKH}$ preparatory to be appended to the *CEL*.

11:    Step 5:    $load[low]=q_{0}$;

12:                    for(j = low+1; j≤up; j++)

13:                    {

14:                        load[j]=load[j−1]−1

15:                    };

16:                    // Initialization on edge load in *CEL*:

17:    Step 6:    while ($n_{pick}\leq N_{d}-q_{0}$)

18:                    //The number of p-nodes picked up does not reach $N_{d}-q_{0}$.

19:                    {

20:                        P = SelectPickupNode (CEL[low~up],e);

21:                        //Select a p-node from *CEL*

22:                        $n_{pick}$ ++;

23:                        // The robot picks up the load at the selected p-node.

24:                        $S_{tabu}$← P;

25:                        // Put p-node P into the taboo list to avoid being selected again.

26:                        Insert ($e_{1}$,*CEL*);

27:                        Insert ($e_{2}$,*CEL*);

28:                        //$e_{1}$,$e_{2}$ are the two adjacent edges connecting p-node P (see Fig 5A).

29:                        up++;

30:                        // A new side has been added to *CEL*.

31:                        $load[e_{1}]=load[e]$;$e_{1}$

32:                        // Edge e is about to be deleted from the *CEL*. Let the edge load of

33:                        the newly added equal to the edge load of edge e before the deletion.

34:                        delete (e,*CEL*);

35:                        copy $L_{LKH}[t]$ to CEL[up];

36:                        // Add a new LKH edge to *CEL*.

37:                        t++;

38:                        $load[e_{2}]=load[e_{1}]+1$;

39:                        // The load of the adjacent edge increases since one load is picked up at P.

40:                        for (*j* = $e_{2}$+1;*j*<up; *j*++)

41:                        {

42:                            load[j]=load[j]+1;

43:                            // The edge load is increased by one in *CEL*.

44:                        }

45:                        load[up]=load[up−1]−1;

46:                        // Load initialization of the edge pointed by up.

47:                        for(j = $e_{2}$; j<up; j++)

48:                        //Check whether the load of edge $e_{2}$ and subsequent edges reach capacity:

49:                        {

50:                            if($load[j]==Q_{\max}$)

51:                            {

52:                            low = *j*+1;

53:                            // If the load on edge j reaches capacity, constrict *CEL*: edge j previous

54:                            and all edges are cut from *CEL*.

55:                            break;

56:                        }

57:                    }

58:                    }        //while

**Algorithm 2.** Selecting a p-node in an ellipse set

1:    Node SelectPickupNode (CEL[low~up])

2:    {

3:        if (e = SelectEdge (CEL[low~up]])

4:        //Use roulette to select edge e in *CEL*.

5:        {

6:            if(P = SelectNode (e)! = true)

7:            //Construct an ellipse set with edge e and get a p-node from it.

8:            {

9:                If there is no p-node in the current ellipse set, then re-select one edge or expand the range of the ellipse set by adjusting parameter R.

10:            }

11:            else return P;

12:        }

13:    }

A feasible solution can be obtained after the end of a loop. If the current path is the shortest one, it will be recorded. The above process is repeated for the specified number of iterations, which is related to both running time and solution quality. In the actual operation, the program [16; 44] and the second phase are connected as a whole.

The running time of the LK algorithm is approximated as $m^{2.2}$ [14], and the complexity of algorithm 1 is m because the number of nodes in the ellipse set is finite. Thus, this extension to the LKH algorithm does not increase the computational complexity of the whole algorithm. The overall complexity of this stage is O ($m^{2}$), where m is the total number of d-nodes.

## 6. Computational experiments

The benchmark instances are from the literature [13]. The depot is placed at (0, 0), and the node property of this node is $q_{0}\;=\;0$. Random 2D coordinates for n-1 sensors were generated in the square ${\left|-500,500\right|}^{2}$ (*n* is the total number of nodes). The proportion of delivery nodes and pickup nodes was set at exactly 1:3, and the mobile robot capacity was set as only a quarter of the total number of sensing holes. The travel cost $x_{ij}$ was the Euclidean distance between node *i* and node *j*.

The benchmark instances can be split into two classes by the number of nodes *n*: small instances with $n\in \left\{20\;,\;30\;,\;40,\;50\;,\;60\;\right\}$ and large instances with $n\in \left\{100\;,\;200\;,\;300,\;400\;,\;500\;\right\}$. A mobile robot departs from the depot with no extra load. Let the robot capacity be $Q_{max}\;=\;25\%\times \left|V_{D}\right|$.

In the following cases, the stopping criterion combines a limit on the total number of iterations (set as 200) and a limit on the computation time (set as 600 s). The limit on the computation time is relevant only for the hardest instances with a large number of nodes and a small vehicle capacity, where the search routines become very time consuming. The algorithm was coded in C++, and all the computational experiments were carried out with an Intel Xeon CPU-E5507 running at 2.27 GHz with 2 GB of memory under Windows 7.

The algorithm updates the *CEL* using three operations (expansion, extension, and constriction), which changes the elements of the *CEL*. For convenience, the algorithm is hereinafter referred to as EEC.

### 6.1 Parameter tuning

There are two parameters in EEC: *M* is the *M*th power of the edge length, which is used in the roulette method to select an edge; and *R* is the scaling factor, which influences the range surrounded by an ellipse. For the same length of the major axis, the larger the value of *R*, the larger the range of the ellipse.

**Fig 8:**
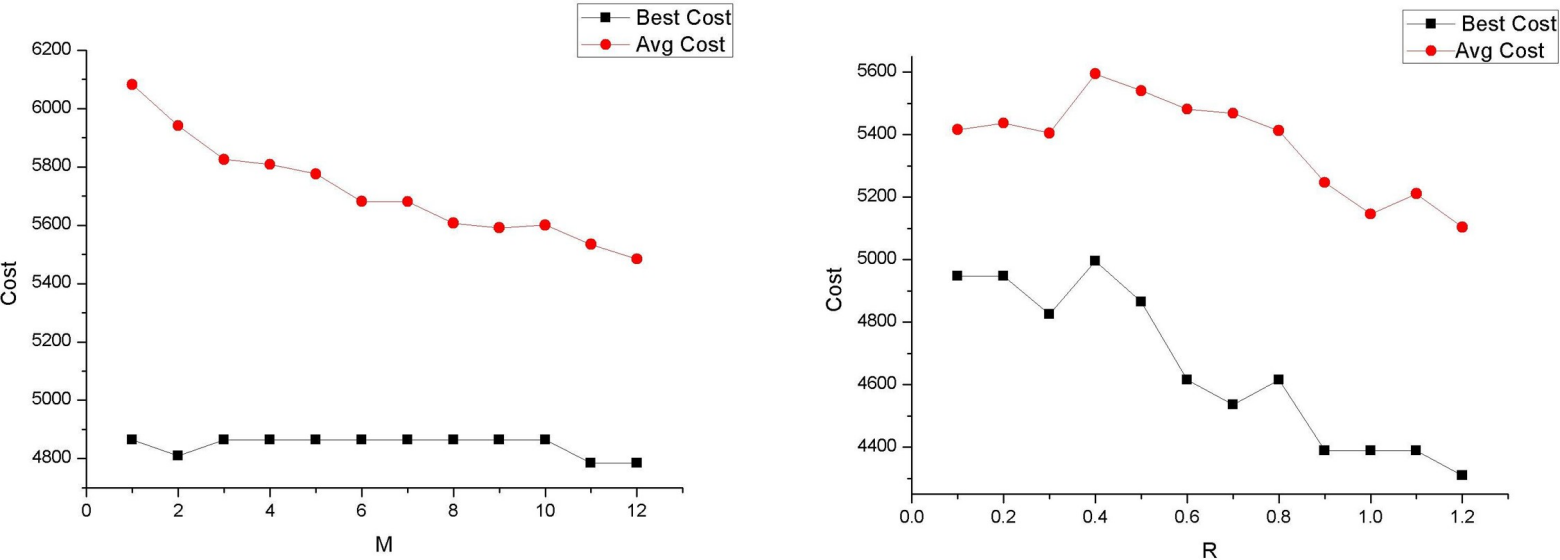
Cost with M and R in 30-node problem (Qmax = 2).

**Fig 9:**
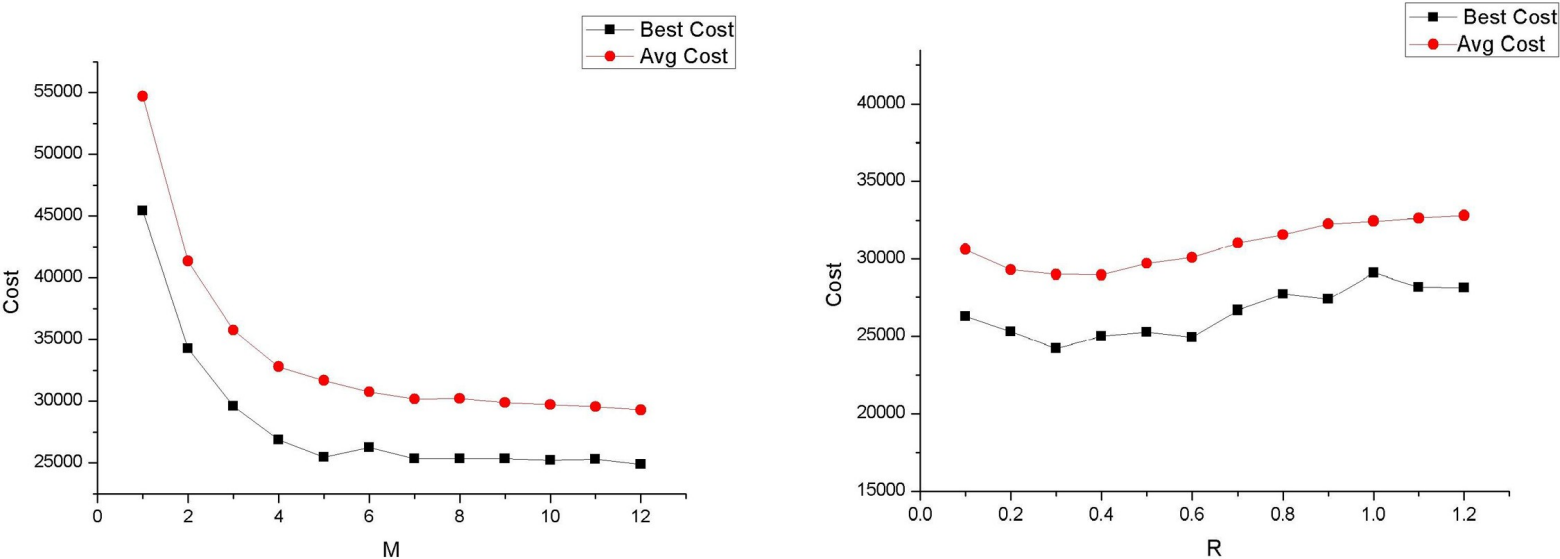
Cost with M and R in a 500-node problem (Qmax = 31).

We run the computer program for 200 iterations over benchmark instances with the following two parameter combinations: *M* ranges from 1 to 12 with step length 2 at *R* = 0.5 (Table 2), and *R* ranges from 0.1 to 1.2 with step length 0.2 at *M* = 10 (Table 3). Figs 8 and 9 show the cost changes with these two parameters for small-scale and large-scale problems, respectively. For *M*, the larger the value, the better the results. When roulette wheel selection is used for the edge selection, the longer the edge, the greater the probability of being selected. However, from this result, a larger *M* may reduce the selection probability of a long edge and increase the selection probability of a shorter edge to avoid search stagnation. However, for *R*, for small-scale problems, the increase in *R* can achieve better results, while for large-scale problems, an increase in *R* does not lead to better results. Moreover, *R* has a greater impact on low-dimensional problems. A possible explanation for this is that because the elliptical area covers fewer p-points for a small-scale problem, parameter *R*, which adjusts the elliptical area, has a greater impact.

**Table 2:** Results under different *M* values (for *R* = 0.5).

n&Q_max_	M	Best Cost	Avg Cost	Time(s)	n&Q_max_	M	Best Cost	Avg Cost	Time(s)
20&1	1	3998.64	3998.64	0.436	100&6	1	11837.3	15907.5	1.216
	3	3998.64	3998.64	0.421		3	11077.1	14231.7	1.107
	5	3998.64	3998.64	0.436		5	11580.7	13670.2	1.092
	7	3998.64	3998.64	0.436		7	11510.6	13290.8	1.076
	9	3998.64	3998.64	0.421		9	11368.1	13171.9	1.076
	11	3998.64	3998.64	0.436		11	11950.6	12970.2	1.076
30&2	1	4864.63	6082.35	0.514	200&13	1	22012.2	26443.1	2.558
	3	4864.63	5825.32	0.499		3	16021.8	19515.4	2.402
	5	4864.63	5775.81	0.468		5	14692.4	17293	2.418
	7	4864.63	5680.89	0.468		7	14641.2	16797.9	2.433
	9	4864.63	5591.04	0.468		9	14672.9	16562.3	2.449
	11	4785.13	5534.49	0.464		11	14713.8	16488.1	2.449
40&3	1	6106.96	7693.28	0.608	300&19	1	29590.6	37375.3	4.492
	3	6196.74	7147.25	0.592		3	18901.3	25073.6	4.034
	5	5943.02	7006.64	0.577		5	17758.7	22035.7	4.131
	7	6088.21	6962.61	0.592		7	17664.8	20961	4.127
	9	6282.15	6946.57	0.561		9	16350.9	20374.2	4.08
	11	6289.31	6962.31	0.577		11	16046.5	20247.9	4.176
50&3	1	6359.55	8878.99	0.74	400&25	1	39163.2	45855.1	6.23
	3	5616.55	8143.36	0.69		3	24379.3	30876.3	5.668
	5	5616.55	8184.5	0.69		5	21740.6	27173.2	5.69
	7	6020.37	8228.56	0.69		7	20838.4	26294.9	5.86
	9	6073.48	8241.16	0.69		9	21951.2	25914.9	5.891
	11	6391.82	8305.98	0.68		11	20828.1	25736.7	5.77
60&4	1	6866.85	9909.5	0.811	500&31	1	45413.3	54711.2	8.367
	3	6393.88	9036.03	0.764		3	29609.3	35757.2	7.81
	5	6673.79	9101.63	0.764		5	25490.3	31680	7.871
	7	7033.62	9192.55	0.748		7	25348.2	30184.8	7.971
	9	7184.18	9202.56	0.748		9	25364	29881.3	8.222
	11	7172.18	9320.44	0.748		11	25311.3	29554.9	7.908

**Table 3:** Results under different *R* values (for *M* = 10).

n&Q_max_	R	Best Cost	Avg Cost	Time(s)	n&Q_max_	R	Best Cost	Avg Cost	Time(s)
20&1	0.1	4717.91	4717.91	0.499	100&6	0.1	11917.9	13755.4	1.279
	0.3	4406.62	4406.62	0.483		0.3	11692	13236.7	1.138
	0.5	3998.64	3998.64	0.421		0.5	11816.7	13001.4	1.107
	0.7	3998.64	3998.64	0.451		0.7	11397.7	13031.2	1.06
	0.9	3512.56	3655.64	0.452		0.9	11404.3	12957.8	1.045
	1.1	3051.21	3387.17	0.421		1.1	11154.6	12699.2	1.06
30&2	0.1	4947.71	5415.61	0.561	200&13	0.1	15989	17869.8	2.668
	0.3	4825.61	5404.59	0.499		0.3	14394.2	15884.2	2.527
	0.5	4864.63	5540.04	0.499		0.5	14710.1	16518.6	2.449
	0.7	4535.32	5468.05	0.546		0.7	14812.3	16884.3	2.34
	0.9	4388.8	5246.62	0.468		0.9	14521.5	17214.0	2.34
	1.1	4388.8	5210.54	0.468		1.1	14169.5	17756.2	2.23
40&3	0.1	6166.21	7028.04	0.67	300&19	0.1	18055.2	21108.7	4.375
	0.3	6084.47	6956.52	0.608		0.3	16773.7	19860.7	4.379
	0.5	6264.36	6984.99	0.577		0.5	16925.7	20288.1	4.023
	0.7	5660.4	6450.07	0.546		0.7	17335.9	21271.8	4.03
	0.9	5362.53	6158.61	0.594		0.9	19728	22334.6	3.825
	1.1	5399.05	6192.65	0.550		1.1	19645.8	22564.5	3.79
50&3	0.1	6944.04	7614.16	0.790	400&25	0.1	22722.1	27261.2	6.066
	0.3	6337.21	8036.07	0.740		0.3	21101.4	25928.8	5.974
	0.5	6461.54	8234.32	0.680		0.5	21724.5	25633.6	5.734
	0.7	5908.66	8161.19	0.640		0.7	23140.4	27112.0	5.606
	0.9	5749.13	8062.7	0.640		0.9	23393.8	27691.5	5.486
	1.1	6251.56	8248.53	0.640		1.1	24616.1	28337.7	5.441
60&4	0.1	7016.19	8521.5	0.858	500&31	0.1	26311.6	30623.8	8.805
	0.3	7068.53	8740.39	0.780		0.3	24204.6	28998	8.334
	0.5	7311.94	9188.17	0.748		0.5	25258.5	29699.2	7.987
	0.7	6705.11	8915.85	0.764		0.7	26670.6	31004.4	7.718
	0.9	7085.72	9055.34	0.764		0.9	27386.2	32249.5	7.695
	1.1	6776.83	8845.02	0.733		1.1	28196.4	32625.9	7.785

### 6.2 Comparison with IMSA and MMAS

MMAS [13] is a type of approach based on the Max-Min Ant System algorithm, whereas IMSA is a simulated annealing meta-heuristic [45]. They have been empirically compared in the literature [13].

Although it is difficult to accurately compare the EEC algorithm proposed in this paper with these two algorithms, we can still list the best and the average results for 10 runs to make some analytical comparisons. The results of the comparison are listed in Table 4. The experimental parameters are as follows: M = 5, R = 0.9, and the number of iterations (cycles) is 150 in a run, whereas MMAS was run for 1000 iterations [13].

**Table 4:** Comparisons of EEC with IMSA and MMAS.

Problem(n/Q)	Algorithms	Best Cost	Avg Cost	Time(s)
20/1	IMSA	**2579.59**	2785.23	20.62
	MMAS	**2579.59**	**2579.59**	37.99
	EEC	3051.21	3387.17	**0.42**
30/2	IMSA	3572.71	3842.03	25.07
	MMAS	**3273.18**	**3416.31**	58.96
	EEC	4308.51	5103.7	**0.468**
40/3	IMSA	4343.67	4752.25	26.77
	MMAS	**3460.33**	**3593.92**	89.41
	EEC	5362.53	6158.61	**0.54**
50/3	IMSA	5211.06	5747.04	33.68
	MMAS	**3933.39**	4093.7	109.12
	EEC	5451.93	8097.26	**0.64**
60/4	IMSA	6432.87	7178.91	34.43
	MMAS	**3741.19**	**4053.19**	137.87
	EEC	6666.42	8768.34	**0.717**
100/6	IMSA	10629.87	11637.17	98.02
	MMAS	**6288.55**	**6619.84**	374.8
	EEC	11142.5	12791.1	**1.06**
200/13	IMSA	23125.5	24302.86	160.89
	MMAS	**10748.05**	**11277**	942.25
	EEC	14169.5	17756.2	**2.23**
300/19	IMSA	37865.17	40627.04	222.16
	MMAS	**15852.06**	21705.81	457.6
	EEC	16104.6	**20166.8**	**4.118**
400/25	IMSA	54204.18	56362.71	331.51
	MMAS	27681	30951.95	551.08
	EEC	**21017.4**	**25737.1**	**5.831**
500/31	IMSA	65559.9	70719.13	397.35
	MMAS	40812.4	45562.97	614.05
	EEC	**24204.6**	**28998.0**	**8.334**

From Table 4, we can see that MMAS requires a much longer running time to achieve the same results as IMSA for maintenance on a global communication structure, while the presented EEC takes far less time than IMSA and MMAS.

**Fig 10:**
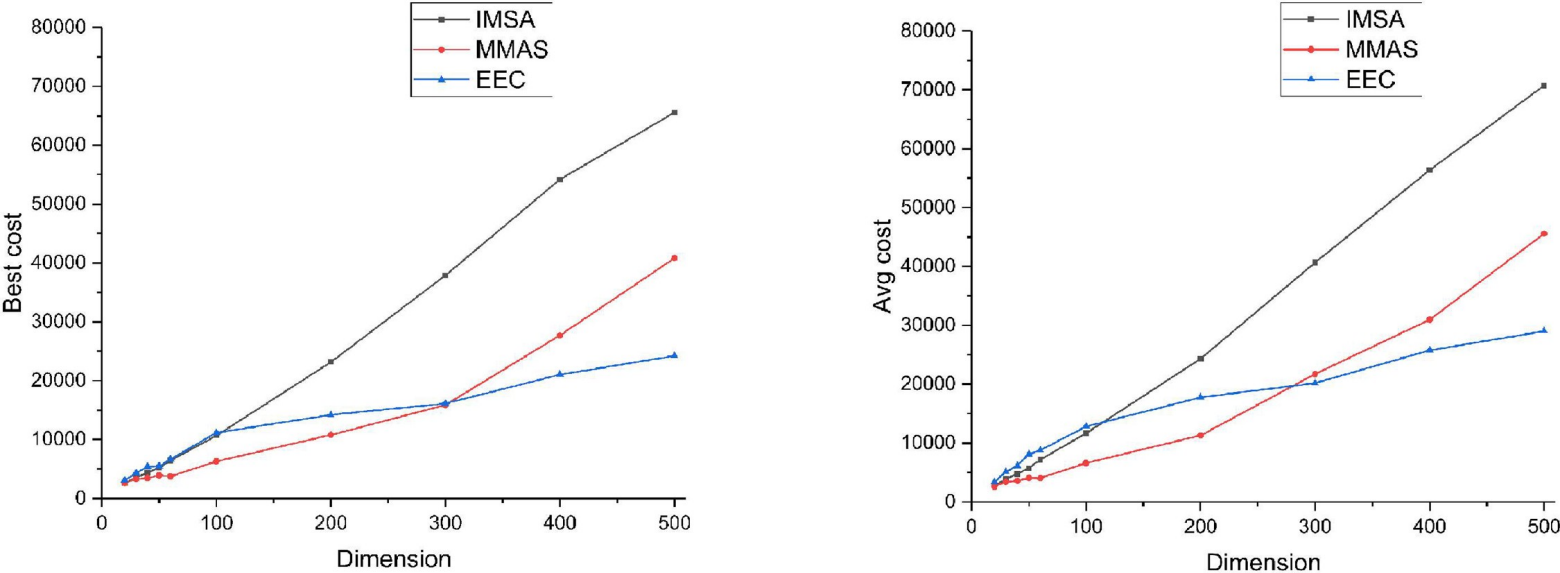
Results of three algorithms with best cost and average cost.

Regarding the quality of the solution, EEC’s performance is poor for small instances, whereas it performs best when the number of sensors exceeds 300. As shown in Table 4, irrespective of the best cost or the average cost, the result of the EEC is significantly better than those of the other two algorithms with only 150 iterations, such as n = 400 or 500. In addition, to further illustrate the advantages of EEC in larger-scale problems, Fig 10 shows their best and average results with the dimensional changes. EEC performs increasingly better when the number of sensors (dimension) becomes larger in the figure which means that EEC has obvious advantages in large-scale cases. The reason may be that too few candidate edges in small instances produce a lack of diversity and make it easy for EEC to fall into a local optimum. For large-scale instances, however, more candidate edges may increase the chances of different edges being selected. In WSNs, the replacement problem is usually large scale. Thus, EEC is more suitable for the application.

## 7. Conclusions

We extended the carrier-based coverage repair problem in wireless sensor and robot networks proposed by Falcon et al. [13] to the application scenario of large-scale sensor replacement. In order to adapt to this large-scale problem, the mature, excellent LKH algorithm is used in the first stage to obtain the optimal LKH cycle to connect damaged sensors. Then, in the second stage, ellipse sets and the *CEL* are designed as candidate sets for selecting edges and nodes. Further, expansion, extension‚ and constriction on the edges are proposed to update the *CEL* with the guidance of the LKH cycle. In comparison with the other two algorithms, the proposed two-stage method reduces the computational time significantly, and the experimental results with 400 and 500 sensors show that the optimization results are much better than those obtained with the compared algorithms.

This algorithm uses only the simple roulette method to select edges and nodes. In future work, higher-level methods may be integrated into it for better results. Moreover, this algorithm should be extended to problems with multi-robots. As a basic method for the one-commodity traveling salesman problem with selective pickup and delivery, this algorithm can be applied not only to the sensor distribution problem but also to industry management or transportation problems for resource assignment.
